# Magnetic Nanoscalpel for the Effective Treatment of Ascites Tumors

**DOI:** 10.3390/jfb14040179

**Published:** 2023-03-24

**Authors:** Tatiana Zamay, Sergey Zamay, Natalia Luzan, Victoriya Fedotovskaya, Albert Masyugin, Fyodor Zelenov, Anastasia Koshmanova, Elena Nikolaeva, Daria Kirichenko, Dmitry Veprintsev, Olga Kolovskaya, Irina Shchugoreva, Galina Zamay, Ivan Lapin, Anna Lukyanenko, Andrey Borus, Alexander Sukhachev, Mikhail Volochaev, Kirill Lukyanenko, Alexandr Shabanov, Vladimir Zabluda, Alexey Zhizhchenko, Aleksandr Kuchmizhak, Alexey Sokolov, Andrey Narodov, Vladimir Prokopenko, Rinat Galeev, Valery Svetlichnyi, Anna Kichkailo

**Affiliations:** 1 Federal Research Center “Krasnoyarsk Science Center” of the Siberian Branch, Russian Academy of Sciences, Krasnoyarsk 660036, Russia; 2 Laboratory for Biomolecular and Medical Technologies, Prof. V.F. Voino-Yasenetsky Krasnoyarsk State Medical University, Krasnoyarsk 660022, Russia; 3 JSC «NPP «Radiosviaz», Krasnoyarsk 660021, Russia; 4 Laboratory of Advanced Materials and Technology, Siberian Physical Technical Institute, Tomsk State University, Tomsk 634050, Russia; 5 L.V. Kirensky Institute of Physics, Siberian Branch of the Russian Academy of Sciences, Krasnoyarsk 660036, Russia; 6 Institute of Automation and Control Processes (IACP), Far Eastern Branch of the Russian Academy of Science, Vladivostok 690041, Russia; 7 Far Eastern Federal University, Vladivostok 690950, Russia; 8 V.P. Astafiev Krasnoyarsk State Pedagogical University, Krasnoyarsk 660049, Russia

**Keywords:** magnetic nanodisks, ascitic tumor, magneto-mechanical therapy, “smart nanoscalpel”, DNA aptamers, apoptosis, necrosis

## Abstract

One of the promising novel methods for radical tumor resection at a single-cell level is magneto-mechanical microsurgery (MMM) with magnetic nano- or microdisks modified with cancer-recognizing molecules. A low-frequency alternating magnetic field (AMF) remotely drives and controls the procedure. Here, we present characterization and application of magnetic nanodisks (MNDs) as a surgical instrument (“smart nanoscalpel”) at a single-cell level. MNDs with a quasi-dipole three-layer structure (Au/Ni/Au) and DNA aptamer AS42 (AS42-MNDs) on the surface converted magnetic moment into mechanical and destroyed tumor cells. The effectiveness of MMM was analyzed on Ehrlich ascites carcinoma (EAC) cells in vitro and in vivo using sine and square-shaped AMF with frequencies from 1 to 50 Hz with 0.1 to 1 duty-cycle parameters. MMM with the “Nanoscalpel” in a sine-shaped 20 Hz AMF, a rectangular-shaped 10 Hz AMF, and a 0.5 duty cycle was the most effective. A sine-shaped field caused apoptosis, whereas a rectangular-shaped field caused necrosis. Four sessions of MMM with AS42-MNDs significantly reduced the number of cells in the tumor. In contrast, ascites tumors continued to grow in groups of mice and mice treated with MNDs with nonspecific oligonucleotide NO-MND. Thus, applying a “smart nanoscalpel” is practical for the microsurgery of malignant neoplasms.

## 1. Introduction

Nowadays, cancer is one of the leading causes of mortality in the working-age population, with a lethal outcome of up to 40% [1,2,3,4]. One of the difficult-to-treat oncological diseases is malignant ascites—a liquid tumor. Pathological fluid with cancer cells accumulates in the abdominal or pleural cavity due to the damage of tumors in the lungs, breast, ovaries, stomach, pancreas, liver, and colon [5,6].

Ascites fluid contains cancer and non-cancer cells and other molecular components, creating a unique microenvironment that modifies tumor behavior. These factors affect tumor cell proliferation, progression, chemoresistance, and evasion of immunity. Tumor cells induce complex immunosuppression, neutralizing antitumor immunity, which leads to the progression of the disease and the ineffectiveness of treatment, provoking a tumor-stimulating environment [7].

After diagnosis, the patient’s treatment is palliative and its choice should consider the potential risks, benefits, and life expectancy [8]. The most common treatment methods for ascites tumors are paracentesis, peritoneovenous shunt, diuretics, surgical reduction of tumor volume, and intraperitoneal chemotherapy [8]. However, these methods are not very effective and are highly toxic. In this regard, developing new therapy methods for ascites tumors remains highly relevant.

In recent years, non-standard nanomedical instruments and technologies, including physical methods of tumor elimination, have been developed [9,10]. The variety of magnetic nanoparticles has become a unique, novel, and effective tool. Magnetic properties allow remote control of motion, vibrations, rotation, absorption, and energy radiation in electromagnetic fields of a specific frequency, strength, and spatial configuration [11,12]. Combining nanotechnologies with molecular biological tools has recently made it possible to obtain a nanoscale surgical instrument—a “smart nanoscalpel”—capable of selective disruption of single tumor cells [13]. A nanoscalpel comprises two components: the first provides selective targeting and the second damages the tumor cell under external forces.

Superparamagnetic nanoparticles (SNPs) exhibit magnetic properties only when a magnetic field (MF) is applied and can destroy malignant tumor cells [9]. Without an MF, NPs have zero magnetic moments. However, SNPs have limited utility for ascites tumors. [14,15]. Their size limits the magnitude of the magnetic response of superparamagnetic nanoparticles required for liquid tumors with floating cells. The efficiency of cell destruction rises with an increase in the SNP’s magnetic moment. At the same time, the growth of the magnetic moment facilitates their aggregation [16]. SNP chemical synthesis is rather challenging due to the relatively low yield and low reproducibility in the quality of the nanoparticles [17].

The search for new concepts has shown that magnetic disks are highly promising structures for the magneto-mechanical destruction of tumor cells. Magnetic nanodisks (MNDs) with high saturation magnetization and the absence of remanence facilitate remote control by an MF and avoid the agglomeration issue. Thus, MNDs can become powerful magneto-mechanical tools for tumor cell elimination [18].

Currently, MND-based methods for magneto-mechanical microsurgery (MMM) are being actively developed [13]. In particular, gold-plated magnetic-vortex microdisks made of iron and nickel alloy have been used to destroy glioblastoma cells in vitro. In these experiments, the death of tumor cells occurred by apoptosis, which was triggered by vibrations of magnetic microdisks in a rotating magnetic field. The targeted delivery of magnetic disks to tumor cells in these experiments in vitro was carried by protein antibodies to IL132R [19]. However, protein antibodies are immunogenic and have low stability in vivo. The immune system quickly recognizes and destroys antibodies in the organism. Thus, their effect on tumor cells will be low. Therefore, other molecular tumor-recognizing ligands are preferable for in vivo utility. DNA or RNA aptamers are promising for the targeted delivery of MNDs because of their low immunogenicity, high stability, and low cost.

Aptamers are synthetic single-stranded DNA or RNA oligonucleotides capable of specific binding to any molecular and cellular targets: proteins, small organic molecules, viral particles, bacteria, antibodies, whole cells, and even tissues [20,21,22]. They are obtained using SELEX technology (systematic evolution of ligands exponential enrichment), which allows targeted selection of the oligonucleotides with high affinity for specified biological targets [23]. After selection, the aptamers’ synthesis is automated, ensuring economic and fast volume production with minimal change from batch to batch. The structural stability of aptamers provides a long storage period. Aptamers can withstand a wide temperature range; their functional tertiary structure is quickly restored after thermal denaturation. Another significant advantage of aptamers is the ease of their chemical modification [24,25].

**Figure 1:**
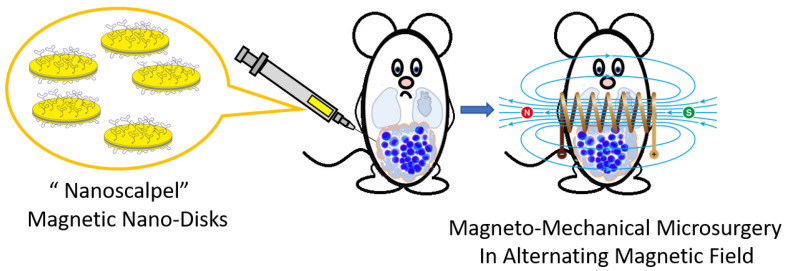
General scheme of tumor MMM. MNDs modified with molecular ligands recognizing tumor cells (“nanoscalpel”) are injected into the body intravenously. After 30 min, sufficient for binding the “nanoscalpel” to tumor cells, the patient is placed in a magnetic coil generating an AMF for 10 min. Oscillations of MNDs, which are associated with membrane or intracellular proteins of tumor cells, trigger the process of tumor cell death in an AMF.

This paper investigates a novel “smart nanoscalpel” tool for microsurgery of intractable ascites tumors. Three-layer MNDs with a quasi-dipole structure (Au/Ni/Au), functionalized by DNA aptamers specific for ascites tumor cells, are remotely controlled by a specific MF. Due to the aptamers, MNDs find and precisely kill tumor cells without damaging healthy ones in a safe alternating MF (AMF) that transforms a magnetic moment into a mechanical one. The general scheme of MMM is shown in Figure 1.

## 2. Materials and Methods

### 2.1. Ethics Statement

This study was carried out in strict accordance with the recommendations in the Guide for the Care and Use of Laboratory Animals of the National Institute of Health. The protocol was approved by the Local Committee on Ethics of Animal Experiments of Krasnoyarsk State Medical University #3 on 16 December 2022. All surgeries were performed under anesthesia, and all efforts were made to minimize animal suffering.

### 2.2. MNDs and Their Functionalization with the Aptamers

Au/Ni/Au MNDs [26] were used in the study. The MNDs were fabricated using the reverse photolithography method (Lift-off process). An EVG101 centrifugation unit was used to apply the photoresist. An EVG610 setup and glass photomasks with a chromium masking layer were used for exposure. The formation of the metallic structure of the nanodisks was carried out by vacuum thermal evaporation in one vacuum cycle.

**Figure 2:**
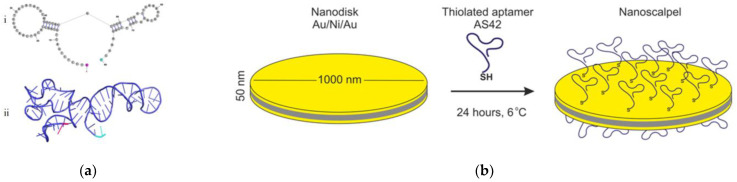
Secondary (**i**) and tertiary (**ii**) structure of the AS42 aptamer. The 5′- and 3′ ends are colored purple and cyan, respectively (**a**). Functionalization of MNDs with oligonucleotides. The yellow layers are aurum, and the gray layer is nickel (**b**).

A thiolated DNA aptamer (AS42) to EAC cells [27] capable of in vivo binding [28] and an oligonucleotide non-specific to EAC cells were used for the MND functionalization. The spatial structure of AS42 was described earlier [29]. MND functionalization (Figure 2) was performed according to the procedure described earlier in [26]. MNDs (Figure 2b) were incubated with 1 µM of thiolated AS42 (AS42-MNDs) or non-specific oligonucleotide (Figure 2a) (NO-MNDs) for 24 h at 6 °C on a shaker to stabilize the disks and prevent their conjugation.

### 2.3. Determining MNDs Concentration

The MND concentration was determined using XRF spectrometry by comparing the X-ray fluorescence intensities for Ni Kα1 = 7.48 keV of the reference sample (C_4_H_6_NiO_4_) of known concentration and the sample under study. Knowing the diameter (900 nm) and the thickness of the disk’s nickel layer (33 nm), we determined the number of MNDs in the sample. The gold coating of the disk in this technique is not significant and is not considered. The XPA spectra were measured on a microfocus X-ray fluorescence spectrometer MS 50 (AMTERTEK, Moscow, Russia).

To increase the sensitivity of XRF spectrometry, a superhydrophobic surface was used in the experiments [30]. The control and test samples were applied and dried on a superhydrophobic substrate. A drop of 20 µL of an aqueous suspension of nanoparticles rushed into the lower part of the concave superhydrophobic mesh and evaporated. During evaporation, the contact area of the droplet with the grid gradually decreases. After complete evaporation of water, all nanoparticles are deposited in a small area ~200 µm in diameter. The creation of a mesh membrane with through holes, along with an increase in the density of nanoparticle deposition from an evaporating drop, made it possible to increase the signal-to-noise ratio and improve the sensitivity of measuring the concentration of deposited nanoparticles when measured using a transmission-type X-ray spectrometer.

### 2.4. Magnetic Coil

The magnetic coil for AMF generating was explicitly developed for this experiment (Figure S1). It consists of Helmholtz rings with an average diameter of 6 cm. The coils are connected to an alternator with a controller system. The coils can generate a magnetic field with a maximum induction of 150 Oe.

### 2.5. Morphological and Magnetic Characterization of MNDs

An electron microscope (Hitachi TM4000, Tokyo, Japan) was used to observe MNDs. A Hitachi HT7700 microscope at the accelerating voltage of 100 kV was used to obtain translucent images of a cross section of a disk on a silicon substrate. Cross-sectional pieces of the samples were prepared using a Hitachi FB2100 (FIB) single-beam focused ion beam system.

The magnetic properties of MNDs were measured with the vibrating sample magnetometer (VSM 8600) Lakeshore (Lake Shore Cryotronics, Westerville, OH, USA). The magnetic structure of MNDs was observed using a scanning magnetic probe force microscope by Veeco MultiMode Nano Scope IIIa SPM System (San Jose, CA, USA) in the mode of formation of magnetic power moment images by the two-pass procedure.

### 2.6. Searching for the Optimal Parameters of the AMF Destructing Tumor Cells with AS42–MNDs

The search for the optimal parameters for the AMF was carried out in vitro on EAC cells isolated from the abdominal cavity of ICR mice with transplanted ascites tumors. Cells were isolated on the 9th day after intraperitoneal EAC transplantation. White, 6-week-old, 25 g, ICR mice were provided by the Shared Core Facilities of Molecular and Cell Technologies at Krasnoyarsk State Medical University, Krasnoyarsk, Russia.

Ascites cells (1 × 10^5^ cells in 100 µL) were incubated with aptamer-modified MNDs for 30 min on a shaker to allow aptamers to bind to EAC cells. Three million AS42–MNDs or NO-MNDs were added to each sample. There were approximately 30 disks per cell in the samples. AMF was generated using the alternating current generator (Figure S1). Cell samples were exposed for 10 min to AMF with the following parameters:

(1) 5, 10, 20, and 50 Hz of a sine-shaped AMF (strength 150 Oe);
(2) 5, 10, 20, and 50 Hz frequencies of a rectangular-shaped AMF (strength 150 Oe) and a duty cycle of 0.5;
(3) A duty cycle of 0.1, 0.2, 0.3, 0.4, and 0.5 at a frequency of 10 Hz of a rectangular-shaped AMF (strength 150 Oe).

Cell disruption was estimated 15 min after the treatment. To assess cell viability after the treatment, treated and untreated EAC cells were stained with trypan blue dye and analyzed using light microscopy (Primo Vert, Carl Zeiss, Hamburg, Germany). Morphological changes in EAC were estimated on the samples that were smeared on glass, stained with Romanowsky–Giemsa dyes, and analyzed using light microscopy (Primo Vert, Carl Zeiss, Hamburg, Germany).

For the fluorescence microscopy, cells’ nuclei were stained with fluorescent DAPI (Sigma-Aldrich, Steinheim, MI, USA), and cell viability was estimated by propidium iodide (Sigma-Aldrich, Saint Louis, MI, USA) dye penetration into the necrotic cells. Staining was performed using the manufacturer’s protocols. Confocal laser scanning microscopy (CLSM) analyses were performed using LSM 780 NLO with an additional channel registering transmitted visible light (Carl Zeiss, Hamburg, Germany) at ×20, 40 magnification; images were processed with ZEN2 software.

To prepare the samples for electron microscopy, droplets with EAC cells bound with the AS42–MNDs were dried on silicon or inverse opals as an absorbent substrate for sample preparation [31]. Then, the cells were fixed with alcohol, washed with deionized water, and dried. Scanning electron microscopy (SEM) was performed using an electron microscope TM4000 (Hitachi, Tokyo, Japan). Images were acquired with the electron beam’s 10 kV and 20 kV voltages.

### 2.7. MMM In Vivo

Here, 25 g, 6-week-old, male, ICR mice with the EAC transplanted intraperitoneally were used as an in vivo ascites tumor model. In 200 µL of saline physiological solution, three million ascites cells were transplanted intraperitoneally into each mouse. The effectiveness of MMM with MNDs was evaluated on day 7 after tumor transplantation.

Three experiments were conducted. The first experiment used a sine-shaped AMF (frequency 20 Hz, MF strength 150 Oe) for MMM. The second and third experiments used a rectangular-shaped AMF (frequency 10 Hz, duty cycle 0.5, and strength 150 Oe). In the first and second experiments, the number of tumor cells and the morphology of ascites cells were evaluated 24 h after MMM. The third experiment evaluated ascites cells’ number and morphology 2 h after MMM.

In all experiments, on the seventh day after tumor transplantation, mice were divided into three groups:

1. The control group without treatment (injected intraperitoneally with 200 µL of phosphate buffer);
2. MMM with NO-MNDs injected intraperitoneally with (69 million MNDs modified with nonspecific aptamers in 200 µL of phosphate buffer per mouse);
3. MMM with AS42–MNDs (69 million MNDs modified with AS42 in 200 µL of phosphate buffer per mouse).

Thirty minutes after injection, the mice were exposed to AMF for 10 min. Then, 2 or 24 h after MMM, the ascites tumor was isolated from the mice. Tumor volume and the number of cells were determined. A hemocytometer was used for the cell counting.

### 2.8. Statistical Analyses

The statistical significance in EAC volumes, ascites cell density, and the total number of ascites cells between the groups was calculated using the nonparametric Mann–Whitney criterion with Bonferroni correction. The differences were considered significant at a level of significance of *p* ≤ 0.05.

## 3. Results

### 3.1. Characterization of Nanodiscs

#### 3.1.1. Structural Characterization of Nanodiscs

**Figure 3:**
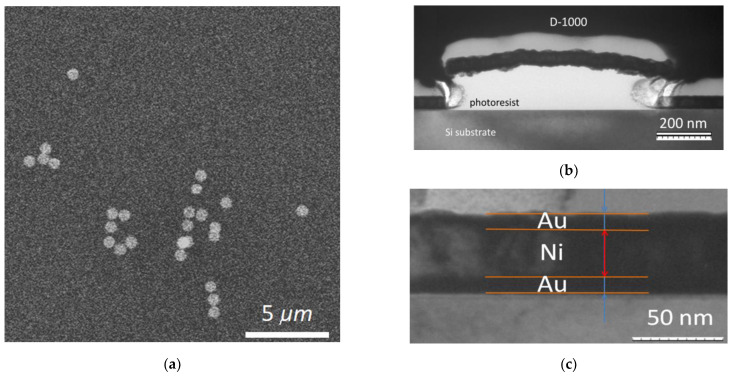
SEM image of MNDs washed off the plate in acetone (**a**) and TEM images of a cross section of a disk on a silicon substrate (**b**,**c**).

The MNDs structures are presented in Figure 3a, where the non-agglomerated disks of an equal diameter of 1 μm are visible. The thickness of the layers was refined using transmission electron microscopy in the cross-section mode (see Figure 3b,c). It can be seen from the TEM data that MNDs are slightly curved. The thickness of the metal layers, counting from below are: Au—11.25 nm, Ni—32.5–34 nm, and Au—5.5 nm. The total thickness of the metal layers of the MND is about 50 nm. The gold layers look darker on a transmission microscope (see Figure 3c).

The magnetic properties of MNDs have been studied previously. It was shown that the magnetic moment of MND in the constant magnetic field of 0.5 T is parallel to its plane [26].

#### 3.1.2. Magnetic Characteristics of MNDs

**Figure 4:**
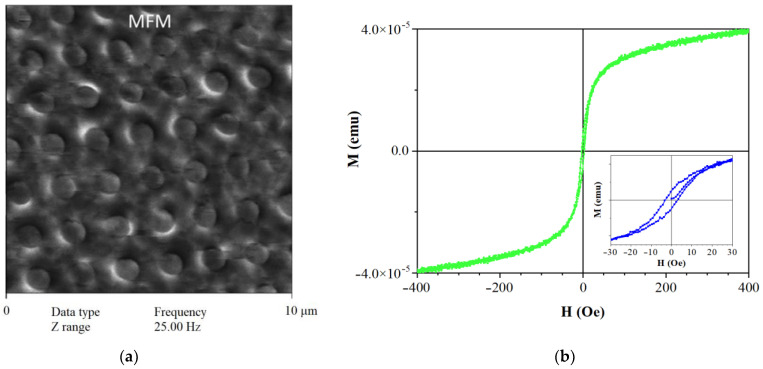
Magnetic force microscopy data (**a**) and room temperature magnetization curves for disks (**b**). The inset shows an enlarged portion of the magnetization curve near zero.

The magnetic properties of MNDs were investigated on samples presented as a dry powder. The MFM results are presented in Figure 4a. They show that the magnetic moments of individual MNDs lie in the disk plane and have a structure close to a dipole one. In this case, the orientation of the MND’s magnetic moments in the image plane is arbitrary.

According to the well-known diagram [32], disks with a diameter of 1000 nm and a magnetic nickel core 30–40 nm thick should have a distribution of magnetization in a plane with an attempt to close the flow at the edges without going out. Therefore, such disks should not have any noticeable magnetic moment. However, for a three-layer AU/Ni/AU microdisk, elastic stresses arise at the edges with a small radius of curvature after fabrication, which lead to a correction of the magnetic parameters. This can potentially contribute to the release of magnetization beyond the disk surface and the emergence of a macroscopic magnetic moment that is observed in the form of light and dark crescent-shaped areas at the boundaries of the image of disks in Figure 4a and indicates their magnetic anisotropy with a quasi-dipole macroscopic moment. In this case, individual nanodisks in the sample’s composition behave like superparamagnetic nanoparticles.

Figure 4b shows the magnetization curve of the studied MND powder. At room temperature, it exhibits superparamagnetic behavior (green curve) with a coercive force of 3-4 Oe (as seen from the blue curve in the inset).

### 3.2. In Vitro Effect of MMM with AS42–MNDs on Ascites Cells

Before the experiments, the concentration of MNDs was measured using XRF analyses (Figure S2). The final concentration of MNDs in the solution for all cell experiments was 1.3 × 10^7^ disks/mL.

The aptamer AS42 was chosen as a specific ligand for targeting EAC cells. The interaction of AS42 functionalized MNDs was estimated using SEM and CLSM. The structure of aptamer AS42 consists of two hairpins connected by one nucleotide. These parts provide the stable conformation of the aptamer in solution since double-helical chains of aptamers are rigid. Moreover, this aptamer has long single-strand parts at the 3′ and 5′ ends (Figure 3a) that can bind with a thiol group without changing overall conformations. The secondary and tertiary structures are presented in Figure 3a. Additional thiolation (for its attachment to the gold surface) did not change aptamer conformation.

**Figure 5:**
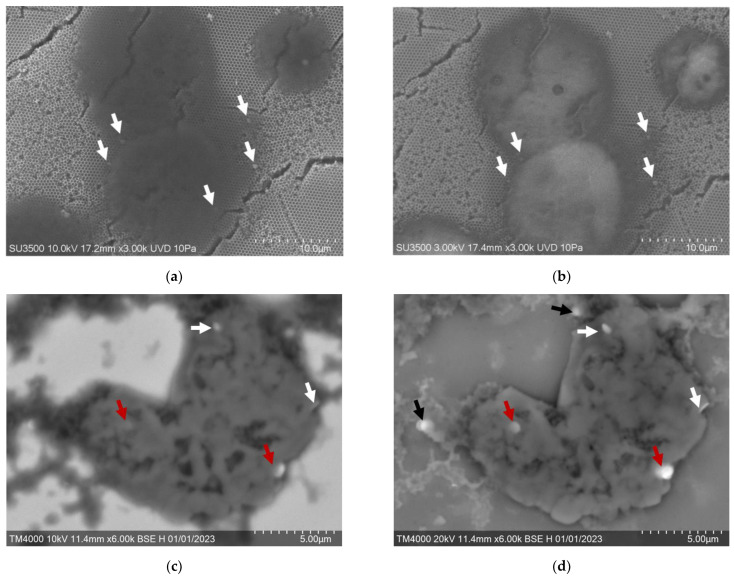

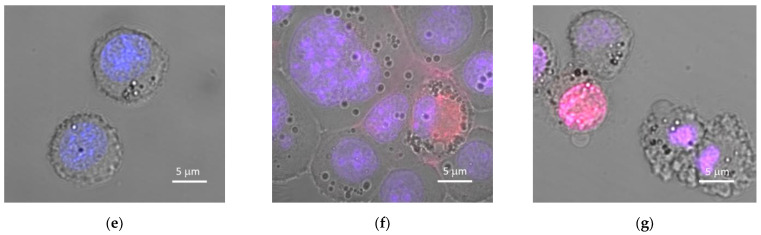
Aptamer-functionalized MND interaction with the target cells. SEM images of ascitic cells bound with AS42–MNDs before (**a**,**b**) and after MMM (**c**,**d**) were recorded in BSE mode (Z-contrast). SEM was performed with a 3 kV–20 kV voltage for the electron beam. CLSM images (**e**–**g**) of ascitic cells after MMM. White arrows indicate MNDs on the surface and black arrows indicate MNDs not bound to the cell, and red show indicate partial penetration. The nucleus is stained with DAPI; propidium iodide penetrates necrotic cells with broken membranes.

EAC cells bound with the AS42–MNDs before exposure to AMF and after MMM were obtained using an SEM at a different accelerating voltage of the electron beam (3 kV to 20 kV) dependent on the sample (Figure 5a–d). Disks on the upper cell surface could be seen at the lower values (Figure 5a,c). Higher accelerating voltages of the electron beam allow one to scan cells in depth (Figure 5b,d). Analysis showed that 1 to 5 MNDs were bound to one ascitic cell. The electron beam of 3 kV voltage visualized MNDs attached to the cell’s upper surface. The higher voltage of 10 kV visualized the disks in depth. However, the thickness of dried cells in the samples did not allow us to estimate whether disks were inside the cell or bound to the membrane.

SEM analysis of cells after MMM showed that one to five MNDs could attach to one cell destructed by AMF. The high voltages of the electron beam (20 kV) made it possible to scan cells in depth. The 10 kV voltage electron beam visualized MNDs attached to the cell’s upper surface. Experiments showed that the MNDs could be located outside the cell membrane (Figure 5c, black arrow) and partially penetrate the cell due to membrane rupture (Figure 5d, red arrow).

Confocal laser scanning microscopy demonstrated that healthy EAC cells (Figure 5e) after AS42–MND-based MMM lost membrane integrity; some of them enlarged (Figure 5f) and underwent necrosis (Figure 5f,g). DAPI stained viable cells’ nuclei (Figure 5e) and became red in necrotic cells because the fluorescence dye propidium iodide penetrated the cell through the broken membrane (Figure 5f,g). The selectivity of the AS42–MND-based MMM has been demonstrated in mice mesenchymal cell cultures. MMM did not influence these cells and did not cause necrosis (Figure S3).

### 3.3. Optimization of Parameters for AS42–MND-Based MMM In Vitro

MMM outcome depends on the AMF parameters; therefore, it is crucial to choose the best parameters for particular MNDs. The effectiveness of tumor cell destruction with AS42–MNDs relies on transforming the disk’s magnetic moment into a mechanical moment. This process causes cell membrane rupture and necrosis or triggers the intracellular signaling apoptosis pathway. The mechanical moment must be sufficient for the transformation. The search for the optimal AMF parameters was carried out on the EAC cells.

**Figure 6:**
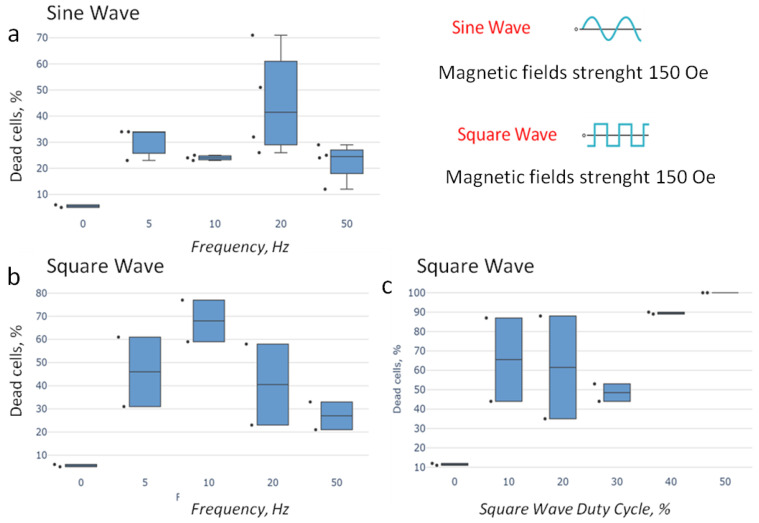
Parameter selection of an AMF for the most effective destruction of EAC cells using MNDs functionalized by oligonucleotides in vitro. Frequency selection with a sinus-shaped and rectangular-shaped AMF, (**a**,**b**), respectively. Duty cycle selection with a rectangular-shaped AMF (**c**).

The first experiment was performed with a sine-shaped AMF. The effect of the AMF frequency, varying from 5 to 50 Hz, is presented in Figure 6. It was determined that the destruction of ascites cells occurs at approximately the same level at a frequency of 5, 10, and 50 Hz. The 20 Hz frequency for a sine-shaped AMF increased the proportion of dead cells after the MMM by a factor of 7. Thus, the optimal parameters for MMM with the sine-shaped field are a frequency of 20 Hz and a strength of 150 Oe. Therefore, these parameters for the AMF were used to evaluate the biological effect of the “smart nanoscalpel” in in vivo experiments.

The influence of a rectangular-shaped AMF was studied with a 0.5 constant duty cycle (Figure 6).

As can be seen in Figure 6, the optimal frequency for the effective destruction of ascites cells is 10 Hz. The proportion of dead cells in the ascites cell sample after AMF exposure increased by more than 11 times. An AMF with a frequency of 20 Hz increased the proportion of dead cells by about 6–8 times compared with a 5 Hz AMF. The 50 Hz AMF frequency was the least effective for destroying ascites cells.

The study of the duty-cycle influence of a rectangular-shaped AMF on the destruction of ascites cells was carried out within variations of this parameter from 0.1 to 0.5. When the duty cycle of a rectangular-shaped AMF was 0.5, all ascites cells died and the death rate reached 100% (Figure 6). Therefore, these parameters were used further to study the effectiveness of MMM with AS42–MNDs in vivo.

### 3.4. Destruction of EAC by MNDs in an AMF In Vivo

The biological effect of MNDs under an AMF in vitro can differ from the effect in vivo conditions. It is necessary to consider the MNDs’ behavior in the organism to achieve the required biological effect. A literature analysis by S. Wilhelm et al. showed that only 1% of nanoparticles injected into the body penetrate solid tumors. Most nanoparticles accumulate in the liver, spleen, and lungs [33,34].

To select the parameters of the AMF that are most effective for tumor destruction in vivo, we used a sine and a rectangular-shaped AMF with the optimal parameters for the destruction of tumor cells. The total volume of the ascites tumor and the density and number of ascites cells assessed the effectiveness of tumor destruction. Ascites cells from mice’s abdominal cavities were collected twice, 2 and 24 h after MMM. The most effective in vitro-determined AMF parameters were used in the in vivo experiment.

**Figure 7:**
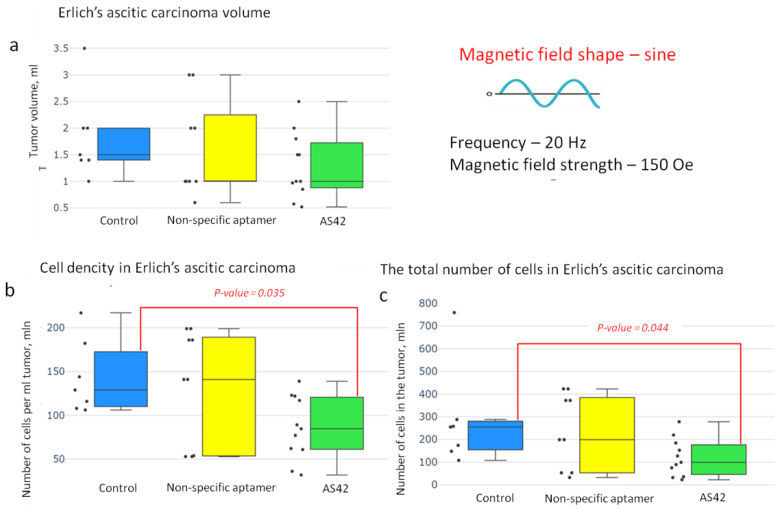
Effectiveness of the “smart nanoscalpel” as an instrument for destroying ascites tumors in vivo using a sine-shaped AMF with a frequency of 20 Hz (24 h after exposure to the magnetic field); (**a**) tumor volume effect, (**b**) cell density effect, and (**c**) the total number of cells in the tumor effect.

The studies showed that with exposure to a sine-shaped AMF, the EAC volume of control mice without the treatment and mice treated with NO-MNDs were almost the same (about 1.5 mL). The tumor volume decreased by 50% when MMM was conducted with AS42–MNDs. However, this decrease was statistically insignificant (Figure 7).

The density of ascites cells in the mouse tumors after the MMM with AS42–MNDs was almost 1.5 times lower than in the other groups. The total number of EAC cells in the AS42–MNDs group was significantly less.

The optimal parameters for the MMM with a square-shaped AMF (10 Hz frequency with a duty cycle of 0.5) were used to evaluate the effectiveness of the “smart nanoscalpel” in vivo 2 and 24 h after MMM.

**Figure 8:**
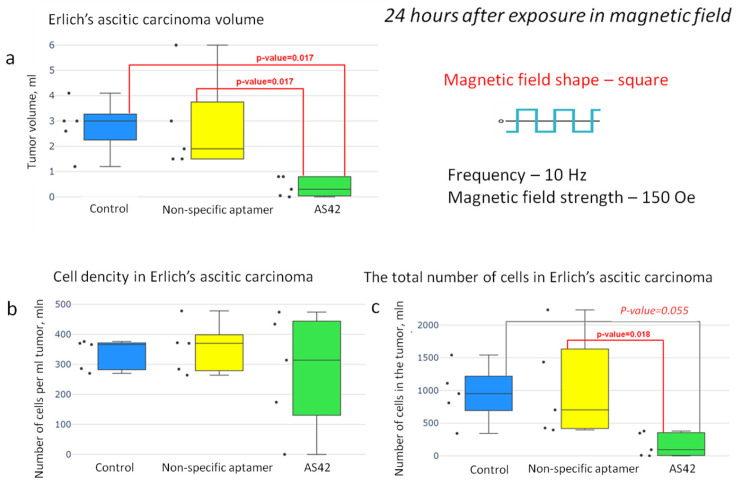
Effectiveness of the “smart nanoscalpel” as an instrument for destroying ascites tumors in vivo using a square-shaped AMF with a frequency of 10 Hz and a duty cycle of 0.5 (24 h after exposure in the magnetic field); (**a**) tumor volume effect, (**b**) cell density effect, and (**c**) the total number of cells in the tumor effect.

The results show that the volume of ascites in animals 24 h after MMM with AS42–MNDs was significantly lower than in the control groups (0.3 mL versus 3 mL in the control groups) (Figure 8). At the same time, the cell density was approximately equal in the tumors of all groups of mice. The number of ascites cells in the mice tumor after MMM with AS42–MNDs was reduced compared with the control.

**Figure 9:**
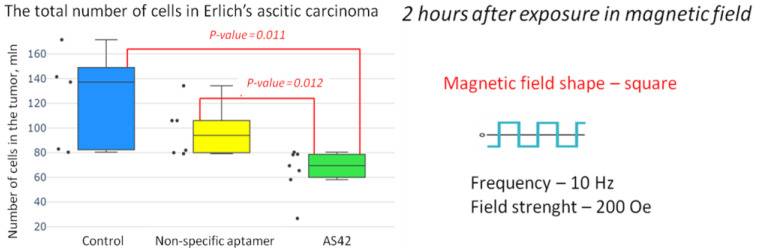
The total number of EAC cells after exposure to the magnetic field in a square-shaped AMF at a frequency of 10 Hz, a duty cycle of 0.5, and a field strength of 150 Oe in vivo.

A third series of experiments was performed to study the tumor’s changes during its destruction. We determined the total number of cells in the tumor and their morphological changes 2 h after the therapy. The number of cells in the tumor treated with MMM and AS42–MNDs decreased significantly (Figure 9). However, a downward trend was observed when treating with NO-MNDs. That can be explained by the nonspecific entry of disks into ascites cells.

The morphology of ascites cells in animals treated with AS42–MNDs differed significantly from those in the control animals (Figure 9). Many cells undergoing apoptosis and necrosis were present in the ascitic fluid. Many cells were destroyed with separate nuclei.

**Figure 10:**
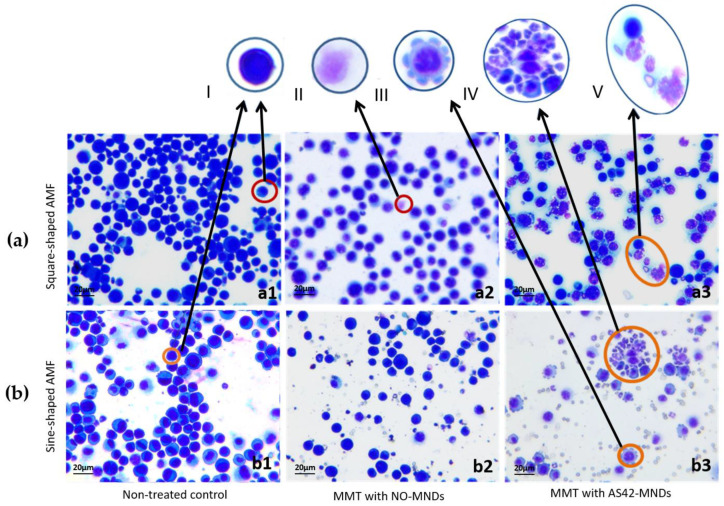
Morphology of EAC cells in mice after MMM in a square-shaped (**a**) and sine-shaped (**b**) AMF at a frequency of 10 Hz, a duty cycle of 0.5, and a field strength of 150 Oe in vivo. Panel (**a1**, **b1**) corresponds to non-treated controls, (**a2**, **b2**) to NO-MNDs, and (**a3**, **b3**) represents cells from AS42–MNDs. I—ascitic cell; II—ascitic cell with the damaged membrane; III—ascites cell in the blebbing state; IV—ascitic cells and apoptotic bodies; V—ascitic cell and cells with the damaged membranes. 20× magnification.

Sine-shaped and square-shaped AMFs led to a significant decrease in the number of ascites cells in EAC. However, these fields differed in their effect on EAC cells in vivo. Figure 10 shows that AS42–MNDs in a square-shaped AMF caused necrosis. Disks connected to ascites cells transformed the magnetic moment into a mechanical one and destroyed the cell membrane. Necrosis led to the release of intracellular contents. Smears obtained from ascitic fluid of mice treated with NO-MNDs also indicate the presence of damaged tumor cells, separate nuclei, and enlarged cells, which is evidence of necrosis. In this case, the MNDs penetrate the ascites cells nonspecifically and destroy them under the influence of a magnetic field, leading to a decrease in the total number of cells in the EAC. Thus, MNDs can be used for MMM even without targeting molecules but with lower effectivity.

The sine-shaped AMF and AS42–MNDs triggered apoptosis processes in the cells (Figure 10). Some of the cells were in a state of initial blebbing, and many apoptotic bodies were observed. Fragments of dead cells are usually quickly (on average in 90 min) phagocytized by macrophages or neighboring cells, avoiding the development of an inflammatory reaction. The morphologically recorded apoptosis usually lasts 1–3 h [35]. Some of the EAC cells of mice treated with NO-MNDs were found in a state of initial blebbing, and a small number of apoptotic bodies were present (Figure 10).

## 4. Discussion

An essential biomedical task is developing a “smart nanoscalpel” consisting of magnetic nanodisks and tumor-recognizing molecules that can selectively destroy tumor cells in an AMF in vivo. Nanodisks with magnetic anisotropy are highly sensitive to magnetic stimuli. Disks acquired high magnetization in a weak external MFs because they have zero total magnetization without a magnetic field.

The targeting of the “nanoscalpel” was achieved by functionalizing MNDs with the DNA aptamer AS42, which were used as bio-identifying molecules. Electron microscopy showed that the MNDs penetrated the ascites cells. Based on the literature, the penetration of magnetic disks into the cell is carried out by endocytosis followed by encapsulation in lysosomes [36]. In this case, it is assumed that the lysosomal membrane rupture under the influence of an AMF is the leading cause of cell death. At the same time, the penetration of disks into the cells without the influence of an AMF does not reduce the viability of the tumor cells [26]. Their penetration begins with the interaction with cell membrane proteins, which are specific targets for the AS42 aptamer.

The search for the optimal AMF parameters for effective tumor destruction showed that a square-shaped AMF with a duty cycle of 0.5 and a frequency of 10 Hz is preferable for a “smart nanoscalpel”. Two hours after MMM, the number of cells in the tumor begins to decrease due to the mass death of ascites cells. It should be considered that intact ascites cells in the tumor continued proliferating. It is well known that the ascites cells double every 24 h. Consequently, one of the most critical challenges in developing a “smart nanoscalpel” for microsurgery of malignant tumors is the problem of selecting the number of nanodisks sufficient to destroy all of the tumor cells.

However, there is a problem of too rapid tumor elimination. The destruction products may be toxic to the organism and pose no less danger than living tumor cells. In studies of the effectiveness of the “smart nanoscalpel”, 69 million MNDs were injected into each animal. That is about two times fewer than EAC cells. Nevertheless, a “smart nanoscalpel” led to a significant reduction of the tumor cells.

The amplitude of the magnetic field of a sinusoidal shape will change smoothly, and, accordingly, this leads to the fact that the oscillations of the magnetic disk during the transformation of the magnetic moment into a mechanical one occur smoothly. Aptamers associated with magnetic disks, when the magnetic moment is transformed into a mechanical one, gently pull on the target protein. Thus, without disturbing the cell membrane, they activate the proteins of signaling pathways leading to apoptosis. With a rectangular-shaped magnetic field, magnetic disks carry out sharper oscillations, which may be the reason for violating the integrity of the cell membrane. Such damage to the cell membrane leads to the release of the cell’s contents to the outside and cell death by necrosis.

Despite the method seeming to be very promising, there are still some limitations connected with MNDs and dead cell elimination from the body. For the internal treatment of ascites or solid tumors, triggering the apoptosis pathway by MMM is preferable. In the case of using MMM as an additional instrument during surgery, the destroyed cells and the disks can be removed by simple washing.

Experiments on ascitic carcinoma showed the fundamental possibility of nanodisks targeting and destroying individual tumor cells. In the future, it is planned to improve this method for the application for the intra-surgical removal of individual tumor cells to prevent tumor recurrence during the resection of solid tumors, followed by washing of disks and destroyed cells. The use of disks for this procedure is primarily because the disks are not biocompatible and due to their large size, they are difficult to remove from the body. Using MNDs intra-surgically is a promising technology for treating malignant tumors since modern technology development makes it possible to obtain disks for tumor therapy on an industrial scale.

An important point in work is that a “nanoscalpel” (AS42–MNDs) in a sine-shaped AMF with a frequency of 20 Hz and a strength of 150 Oe causes cell death by apoptosis. In comparison, a square-shaped field with a frequency of 10 Hz, a strength of 150 Oe, and a duty cycle of 0.5 “nanoscalpel” destroys cellular membranes and causes necrosis.

## 5. Conclusions

Here we present the discovery of physical characteristics of magnetic nanodisks with a quasi-dipole three-layer Au/Ni/Au structure with a size of 920 ± 10 nm and their application for magneto-mechanical microsurgery. A unique alternating current generator was developed for the experiments. The most effective MMM parameters were determined in vitro and applied in vivo for effective ascites tumor cell destruction. Thus, MMM with the “nanoscalpel” remotely controlled by an alternating magnetic field may become one of the most effective methods for ascites tumor therapy.

## Data Availability

The data presented in this study are available on request from the corresponding author.

## Supplementary Materials

The following supporting information can be downloaded at: https://www.mdpi.com/article/10.3390/jfb14040179/s1, Figure S1: AMF generating coil for MMM with magnetic MNDs. Structural-functional scheme of an alternating current generator (a) and its utility in vivo and in vitro; Figure S2: XRF analyses of MNDs number in the sample. (a) The spectrum of a dried on superhydrophobic surface 20 μL aqueous solution of the MNDs. (b) The spectrum of a dried on superhydrophobic surface 20 μL aqueous solution of 12 mg/mL C_4_H_6_NiO_4_; Figure S3: AS42–MND-based MMM did not cause necrosis in mice mesenchymal cells. CLSM image of cells incubated with AS42–MNDs before (a) and after (b) MMM. Propidium iodide penetrates necrotic cells with the broken membranes.
