# Supplementary material for: Magnetic Nanoscalpel for the Effective Treatment of Ascites Tumors
Source: J Funct Biomater. 2023 Mar 24;14(4):179. doi: 10.3390/jfb14040179 (PMC10145876; doi:10.3390/jfb14040179)
Supplement: Supplementary file 1 [file jfb-14-00179-s001.zip › jfb-2288137-supplementary.pdf]

Journal of Functional Biomaterials

Supplementary

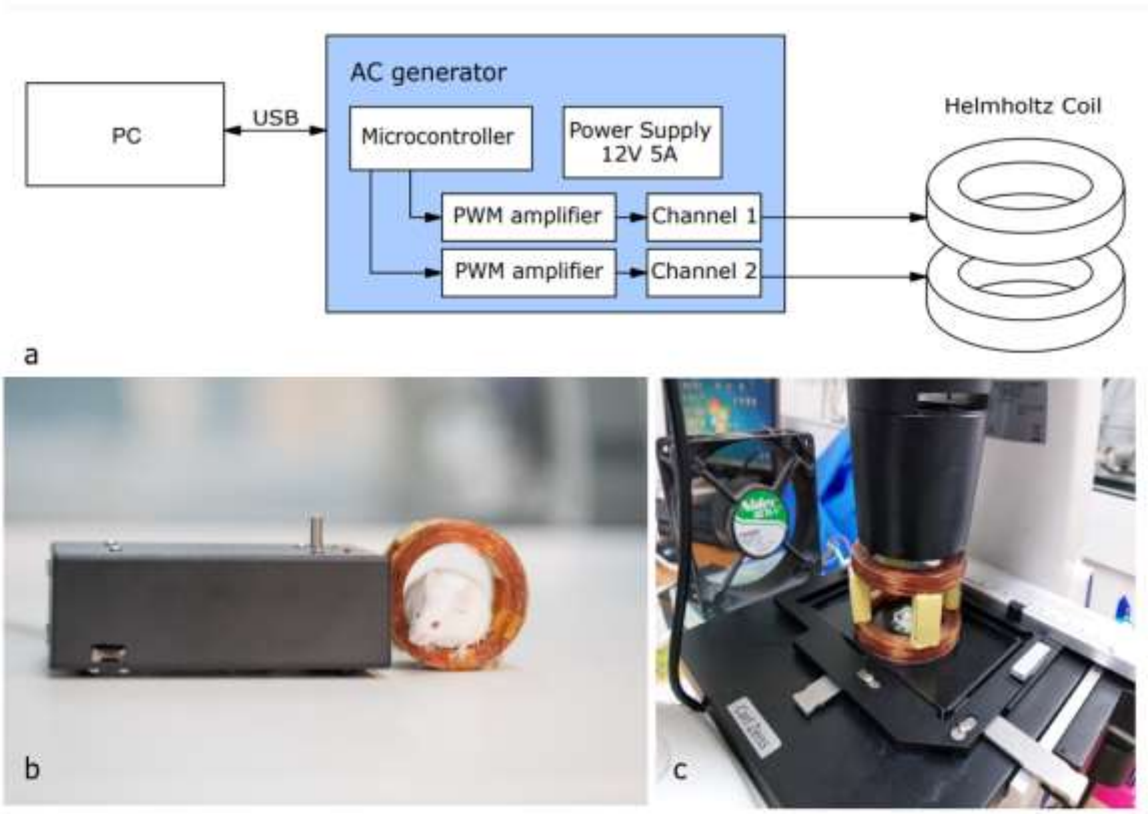

Figure S1. AMF generating coil for MMT with magnetic MNDs. Structural-functional scheme of an alternating current generator (a) and its utility in vivo and in vitro.

a

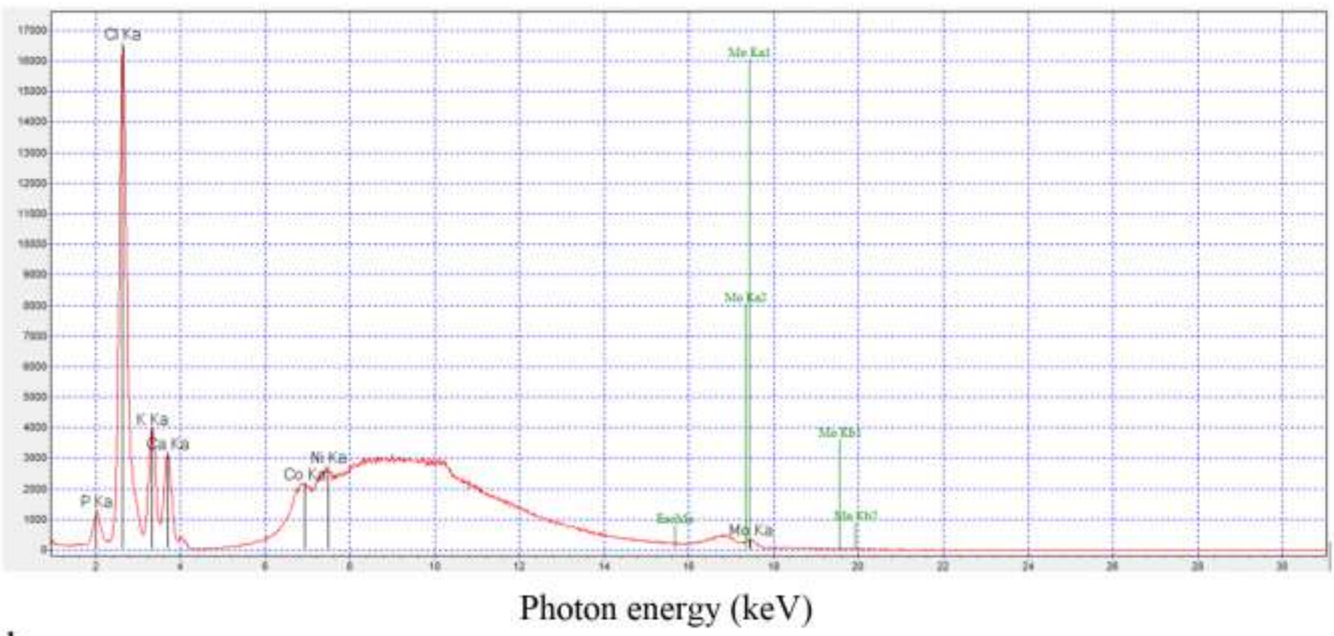

b

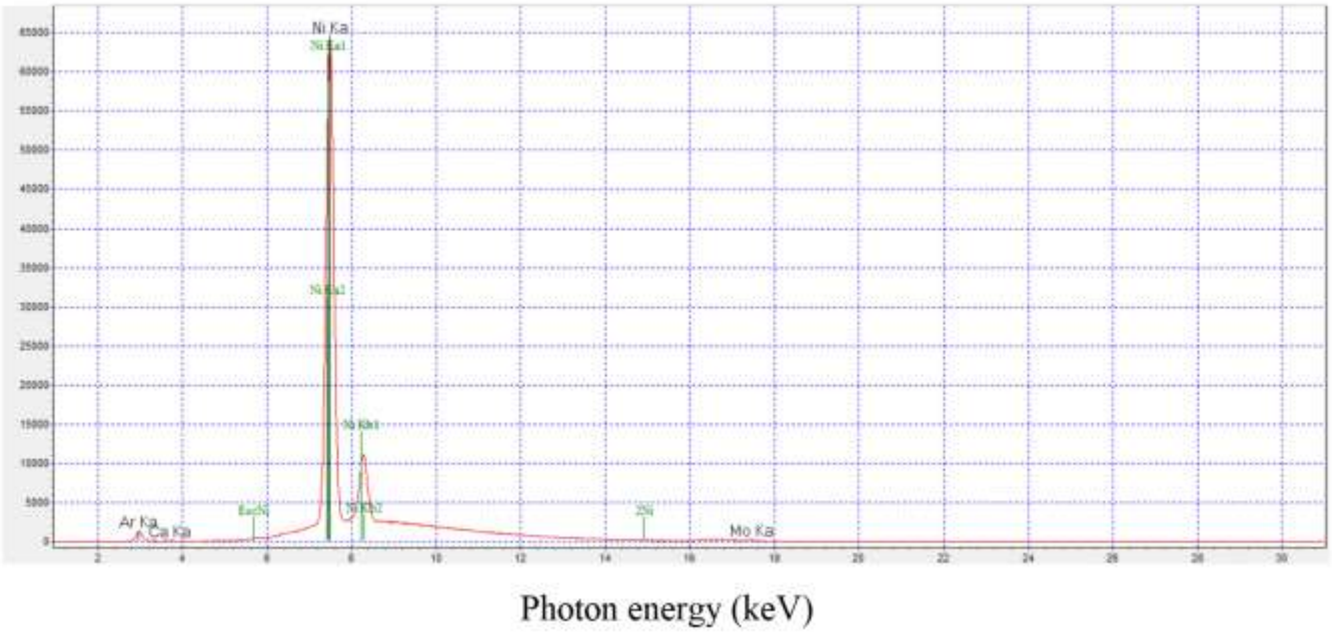

Figure S2. XRF analyses of MNDs number in the sample. (a) Spectrum of a dried on super hydrophobic surface 20 µl aqueous solution of the MNDs. (b) Spectrum of a dried on super hydrophobic surface 20 µl aqueous solution of 12 mg/mL C4H6NiO4.

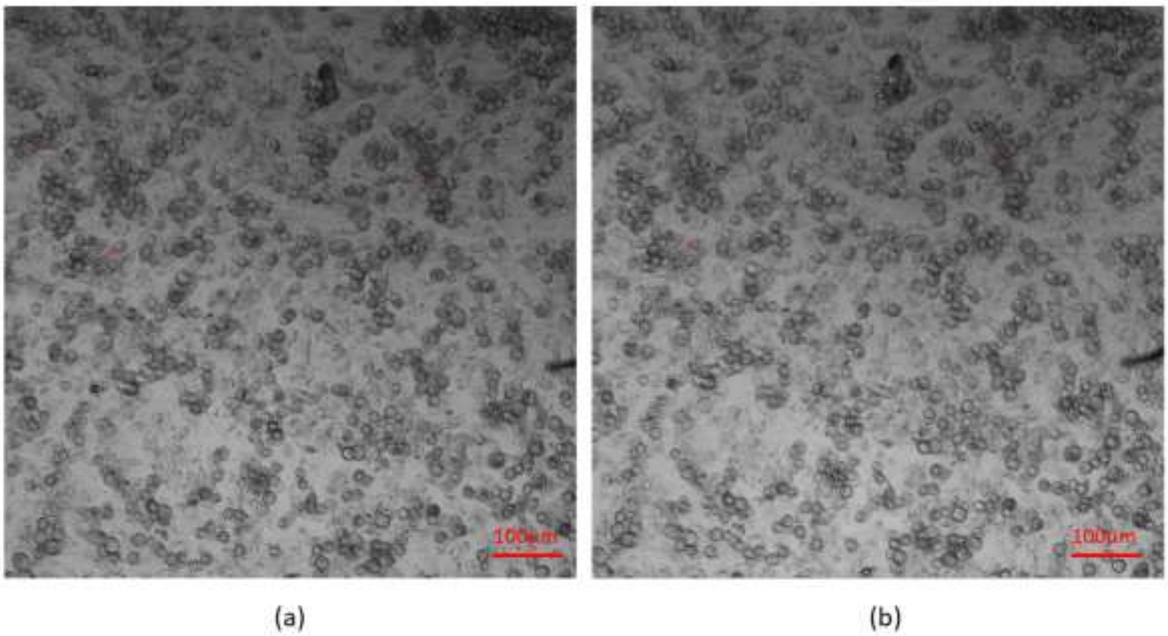

Figure S3. AS42-MNDs-based MMM did not cause necrosis in mice mesenchymal cells. CLSM image of cells incubated with AS-42-MNDs before (a) and after (b) MMM. Propidium iodide penetrates necrotic cells with the broken membranes.
